# Independent of ErbB1 gene copy number, EGF stimulates migration but is not associated with cell proliferation in non-small cell lung cancer

**DOI:** 10.1186/1475-2867-13-38

**Published:** 2013-04-30

**Authors:** Camila Lauand, Paula Rezende-Teixeira, Beatriz Araújo Cortez, Evandro Luís de Oliveira Niero, Gláucia Maria Machado-Santelli

**Affiliations:** 1 Department of Cell and Developmental Biology, Institute of Biomedical Sciences, University of Sao Paulo, Av. Prof. Lineu Prestes, 1524, Butantã, São Paulo, SP 05508-000, Brazil

**Keywords:** Epidermal growth factor receptor, Lung cancer, Epidermal growth factor, Proliferation, Tyrosine kinase inhibitor, Cell migration

## Abstract

**Background:**

Lung cancer often exhibits molecular changes, such as the overexpression of the ErbB1 gene. ErbB1 encodes epidermal growth factor receptor (EGFR), a tyrosine kinase receptor, involved mainly in cell proliferation and survival. EGFR overexpression has been associated with more aggressive disease, poor prognosis, low survival rate and low response to therapy. ErbB1 amplification and mutation are associated with tumor development and are implicated in ineffective treatment. The aim of the present study was to investigate whether the ErbB1 copy number affects EGFR expression, cell proliferation or cell migration by comparing two different cell lines.

**Methods:**

The copies of ErbB1 gene was evaluated by FISH. Immunofluorescence and Western blotting were performed to determine location and expression of proteins mentioned in the present study. Proliferation was studied by flow cytometry and cell migration by wound healing assay and time lapse.

**Results:**

We investigated the activation and function of EGFR in the A549 and HK2 lung cancer cell lines, which contain 3 and 6 copies of ErbB1, respectively. The expression of EGFR was lower in the HK2 cell line. EGFR was activated after stimulation with EGF in both cell lines, but this activation did not promote differences in cellular proliferation when compared to control cells. Inhibiting EGFR with AG1478 did not modify cellular proliferation, confirming previous data. However, we observed morphological alterations, changes in microfilament organization and increased cell migration upon EGF stimulation. However, these effects did not seem to be consequence of an epithelial-mesenchymal transition.

**Conclusion:**

EGFR expression did not appear to be associated to the ErbB1 gene copy number, and neither of these aspects appeared to affect cell proliferation. However, EGFR activation by EGF resulted in cell migration stimulation in both cell lines.

## Introduction

Lung cancer is the most common type of cancer in men and women worldwide, and it is the main cause of cancer-related death in the United States, Europe, Japan and China [1-5]. Lung cancer is usually classified as small cell lung cancer (10-15% of cases, associated with smoking), non-small cell lung cancer [(85-90% of cases) also classified as squamous cell carcinoma, adenocarcinoma or large cell carcinoma], or carcinoid tumors of the lung (less than 5% of cases) [6].

Lung cancer cells frequently manifest alterations in their molecular genetics, including changes in epidermal growth factor receptor (EGFR) expression. EGFR is a receptor encoded by the ErbB1 gene, which is located in the 7p12 region of chromosome 7. EGFR is a 170 kDa transmembrane glycoprotein, which contains an extracellular domain, a transmembrane region and a tyrosine kinase domain in the cytosol [7,8]. EGFR is activated by its ligands (EGF, TGF-α, amphiregulin, betacellulin, heparin or epiregulin) that bind to the extracellular domain, causing a conformational change and promoting homo- or hetero-dimerization. This results in the autophosphorylation of the tyrosine kinase domain, thereby initiating an intracellular signaling cascade [9]. After activation, EGFR is internalized and promptly degraded by lysosomes or recycled to the membrane [10].

EGFR phosphorylation can activate two major cytoplasmic signaling routes: RAS and PI-3 kinase/Akt. RAS activation is responsible for inducing a phosphorylation cascade, leading to the activation of MAPKs, ERK1 and ERK2, which regulate the transcription of molecules related to cell proliferation [11,12]. The signaling cascade mediated by PI-3 kinase/Akt is responsible for maintaining cell survival [13]. However, there are additional signals affected by EGFR phosphorylation, for example, the activation of the transcription factor STAT. In addition to proliferation and survival, EGFR can promote other phenomena, such as migration, invasion, differentiation, inhibition of apoptosis and angiogenesis [14]. The activation of EGFR by EGF can also induce epithelial-mesenchymal transition (EMT), which usually corresponds to increased cell motility [15].

EGFR has been associated with disease aggressiveness, poor prognosis, low survival rate, low response to therapy and resistance to cytotoxic agents in some types of tumors [16]. Considering these factors, the development of drugs that target this protein, i.e., EGFR inhibitors, for use in the treatment of lung cancer is gaining importance. ErbB1 gene mutations and amplification usually occur together, and lung cancer cell lines that manifest both events exhibit high levels of EGFR [17,18]. Amplification, mutation and overexpression have been used as biomarkers to select patients for treatment with EGFR inhibitors.

The first inhibitors clinically available for the treatment of non-small cell lung cancer, such as erlotinib and gefitinib, block the tyrosine kinase domain by competing with the ATP-binding site [19]. Humanized monoclonal antibodies that compete with the EGFR-binding sites are being evaluated, for example, cetuximab [19]. Certain mutations in exons of the ErbB1 gene can interfere with the effects of these EGFR inhibitors [20]. The response to EGFR inhibitors depends on the type of EGFR mutation, and many studies have shown the relationship between mutations and EGFR inhibitors [21-24].

The relationship between changes in the ErbB1 gene copy number and EGFR overexpression remains controversial. The impact of each of these changes on cell biology and tumor progression also requires further study. Studies focusing on EGFR and ErbB1 in lung cancer cells are required for a better understanding of the functions of EGFR in tumor biology and for the development of new drugs and new treatment guidelines.

In this study, we aimed to elucidate the relationship between ErbB1 gene copy number and EGFR expression in two cell lines derived from non small cell lung cancer. The number of ErbB1 gene copies was not directly correlated with the EGFR expression. The activation of EGFR by EGF was detected by p-EGFR presence in both cell lines and did not promote cell proliferation when compared to unstimulated cells. However, morphological alterations and increased cell migration were observed and the relation of these alterations with EMT was analyzed.

## Methods

### Cell culture

The human non-small cell lung cancer A549 cell line was obtained from ATCC, and the HK2 cell line was established in our laboratory [25]. Both cell lines were cultured in a 37°C humidified incubator in an atmosphere of 5% CO_2_ and maintained in Dulbecco’s Modified Eagle’s Minimum Essential Medium (DMEM, Sigma, CA, USA) supplemented with 10% fetal calf serum (FCS), (Cultilab, Sao Paulo, Brazil). The AG1478 was purchased from Merck, and the concentration used was 5 μM. The EGF was purchased from Sigma-Aldrich, and the concentrations used were 100 ng/mL and 200 ng/mL. The experimental protocols were approved by the Ethics Committee of the Institute of Biomedical Science, University of Sao Paulo, Brazil (Protocol CEP-ICB n. 389/10).

### Fluorescent in situ hybridization

Slides containing A549 cells were washed for 5 minutes in 2×SSC solution and 1 minute in deionized water. Next, the slides were dehydrated in 70%, 90% and 100% ethanol for 1 minute each, and the sections were air-dried. Ten microliters of ZytoVision SPEC EGFR/CEN 7 Dual Color probe was added, and the samples were covered with a coverslip. The coverslips were sealed with glue. Next, the slides were denatured at 75°C for 5 minutes on a hotplate and transferred to a humidified chamber and hybridized overnight at 37°C. The next day, the glue was removed and the coverslips submerged in 1× Wash Buffer A at 37°C for 1 minute. The slides were washed twice in 1× Wash Buffer A for 5 minutes at 37°C. After this, the slides were washed in PBSA for 1 minute and incubated with TOPRO-3 in a humidified chamber for 20 minutes. The excess TOPRO-3 was removed by washing the slides in PBSA. Antifade Solution was added to the slides, and the slides were covered with coverslip and sealed. The images were obtained using a confocal laser-scanning microscope (LSM 510 – ZEISS).

### Immunofluorescence

The cells were cultured on coverslips in 35 mm dishes containing an initial inoculum of 3 × 10^4^ cells. The preparations were washed with phosphate-buffered saline (PBSA) and fixed with 3.7% formaldehyde for 30 minutes. The cells were washed with PBSA again and permeabilized with 0.5% Triton X-100 for 10 minutes. After this time, the cells were washed with PBSA, and the primary antibodies were added. The incubations with antibodies were performed in a humidified chamber overnight. The antibodies used were as follows: EGFR (Santa Cruz 1:20), p-EGFR (Abcam 1:100) and anti-golgin (Molecular Probes 1:50). The cells were washed with PBSA again, and the secondary antibody (FITC or TRITC) was added and incubated for 3 hours. The nuclei were stained with propidium iodide, and actin was highlighted with phalloidin-FITC. The coverslips were mounted on slides using Vectashield Antifade (Vector, Burlingame, CA, USA). The images were obtained using a confocal laser-scanning microscope (LSM 510 – ZEISS).

### Immunoblots

The medium was removed after the treatments, and the cells were washed with PBSA and lysed with RIPA buffer [50 mM Tris–HCl (pH 7.5), 150 mM NaCl, 0.1% NP-40, 0.5% sodium deoxycholate, 1 mM EDTA and 2 mM EGTA] containing 1 mM sodium orthovanadate, 1 mM phenylmethylsulfonyl fluoride, 10 mg/ml leupeptin and 10 mg/ml aprotinin. The lysates were centrifuged at 20000 × g for 5 minutes. The supernatants were collected, and a BCA assay (Pierce Biotechnology, Rockford, USA) was used to quantify the total protein concentration. Thirty micrograms (30 μg) of protein from the total cell lysates was fractionated by SDS-PAGE on a 10% gel, and the separated proteins were transferred to a PVDF membrane (Amersham Bioscience) (100 V, 250 mA, for 2 hours). A blocking solution (TBS, 5% milk and 0.05% Tween 20%) was added to the membrane for 1 hour. The membranes incubated with antibodies for the detection of phosphorylated proteins were subjected to blocking with 5% BSA (containing the phosphatase inhibitors NaF and orthovanadate). After blocking, the membranes were incubated overnight with an anti-EGFR polyclonal antibody (Santa Cruz) diluted at 1:300 and anti-p-EGFR (1:1000) antibody. A β-Tubulin or β-actin antibodies (1:1000) was used as a control to confirm the loading conditions of the proteins in the gel. The membranes were washed with TTBS (2 × 10 minutes) and TBS (2 × 10 minutes). The immune complexes were detected using peroxidase-conjugated antibodies to mouse or rabbit immunoglobulin (added for 1 hour at room temperature) followed by exposure using the ECL Western blotting detection kit (Amersham Pharmacia). The ImageJ program was used for the densitometric analyses.

### Treatments and number of cells

The cells were cultured in 35 mm dishes with an initial inoculum of 2 × 10^4^ in medium supplemented with 2.5% FCS. After 24 hours, the medium was changed, and different concentrations of FCS, EGF (100 ng/ml; 200 ng/ml) or AG1478 (5 μM) were added. The experimental groups were as follows: control cells (10% FCS), starved control cells cultured with 2.5% FSC (C2.5%FCS), cells stimulated with EGF in 2.5% FCS medium (2.5%FCS + EGF), cells treated with AG1478 in the 2.5% FCS medium (2.5%FCS + AG1478), cells treated with AG1478 and stimulated with EGF after 1 hour (2.5%FCS + AG1478 + EGF) and cells cultured in 10% FCS medium containing EGF (10%FCS + EGF). The cells were maintained in these conditions for 24, 48 and 72 hours. After this period, the cells were washed three times with 500 mL PBSA. After removing the PBSA, we added 500 mL trypsin and inactivated this enzyme with 500 mL of medium. The cells were quantified by counting in flow cytometer (Guava EasyCyte Mini).

### Cell cycle

The A549 and HK2 cells (3×10^4^ cells/35×11 mm dishes) were incubated for 24, 48 and 72 hours with the treatments. The cells were harvested and fixed with 75% ice-cold methanol at 4°C for 1 h. Cells were then washed with PBSA and suspended in propidium iodide staining solution containing 200 μL of PBSA, 20 μL of ribonuclease (10 mg/mL) and 20 μL of propidium iodide (10 μg/mL). The cell suspensions were incubated for 1 h at 4°C, and 5000 cells were analyzed by flow cytometry in each group (EasyCyte Mini - Guava Technologies).

### Mitotic index

After the treatments, the cells were fixed with the same reagents described in the immunofluorescence section. The nuclei were stained with propidium iodide for 15 minutes, and the coverslips were mounted on slides with Vectashield Antifade. The frequency of metaphases was determined in the preparations using a confocal laser-scanning microscope; 2000 cells per slide were counted.

### Real time RT-PCR

The RNA extraction was performed using ChargeSwitch Total RNA Cell Kits. The RNA was quantified using a NanoDrop ND1000 Spectrophotometer. The expression levels were determined by real time RT-PCR analysis (Corbett Research - Rotor Gene 6000 real-time cycler) using an AgPath-ID One-Step RT-PCR kit (Ambion) and SYBR Green (Invitrogen). The qRT-PCR conditions were as follows: 45°C for 10 minutes, 95°C for 15 minutes and 40 cycles [95°C for 15 seconds; Tm°C for 20 seconds; 72°C for 30 seconds] following the melt. The primers used were the following: qEGFR_Right TCCTTTGGGGCATAGATCAG and qEGFR_Left GCTGACCTGGAGGGAACATA (Tm 52°C), qVim_Right TCCAGCAGCTTCCTGTAGGT and qVim_Left GAGAACTTTGCCGTTGAAGC (Tm 55°C), qCyto18_Right GAGCTGCTCCATCTGTAGGG and qCyto18_Left CACAGTCTGCTGAGGTTGGA (Tm 55°C), qEcad_Right AAAGTGATGACCTCCCATGC and qEcad_Left TACCTGCTCACGTCAAATGC (Tm 55°C). The normalization was done against total RNA [26,27].

### Wound healing assay

For the wound healing assay, the A549 and HK2 cells (1×10^5^ cells/35×11 mm dishes) were seeded and incubated for 24 hours at 37°C and then treated according our treatment schedules (C2.5%FCS, 2.5%FCS + EGF, 2.5%FCS + AG1478). After achieving confluence, the cellular layer in each plate was scratched using a plastic pipette tip. The migration of the cells at the edge of the scratch was analyzed at 0, 24 and 48 hours, when microscopic images of the cells were captured. The images were analyzed by qCMA software [28].

### Time-lapse microscopy

For time-lapse experiments A549 and HK2 cells were culture (5×10^4^ cells) in glass bottom dishes with 4 compartments. After 24 h cells in each compartment were culture in one of following conditions: culture medium with 2.5% FCS plus 100 ng/mL of EGF, culture medium with 2.5% FCS plus 5 μM of AG1478, culture medium with 2.5% FCS and culture medium with 10% of FCS. Cells were observed for 12 h in a Biostation microscope (Nikon), which maintained the temperature at 37°C and atmosphere with 5% of CO_2_. Images were taken every 15 minutes using a 20× objective, and route and velocity of 30 cells of each group were determined by MTrackJ plugin of ImageJ software.

### Statistical analysis

The statistical analyses were performed using Minitab 15 software. The tests to determine the difference between the control groups and treatments were as follows: *one-way* ANOVA, *two-way* ANOVA (multiple comparisons by Tukey) and *Student’s t-test*. The differences were considered significant at p ≤ 0.05.

## Results

### Lung cancer cell lines differ in the number of ErbB1 gene copies and levels of EGFR expression

**Figure 1:**
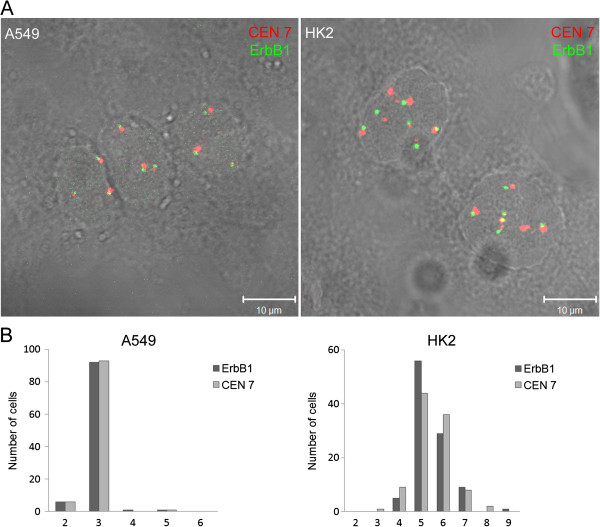
**Quantification of ErbB1 gene copy number in A549 and HK2 cell lines.** (**A**) The presence of centromere 7 (red) and ErbB1 gene (green) was visualized by fluorescence *in situ* hybridization. The nuclei were visualized by interference contrast (DIC). (**B**) The copy number of ErbB1 and centromere 7 per nucleus. One hundred cells of each strain were analyzed.

**Figure 2:**
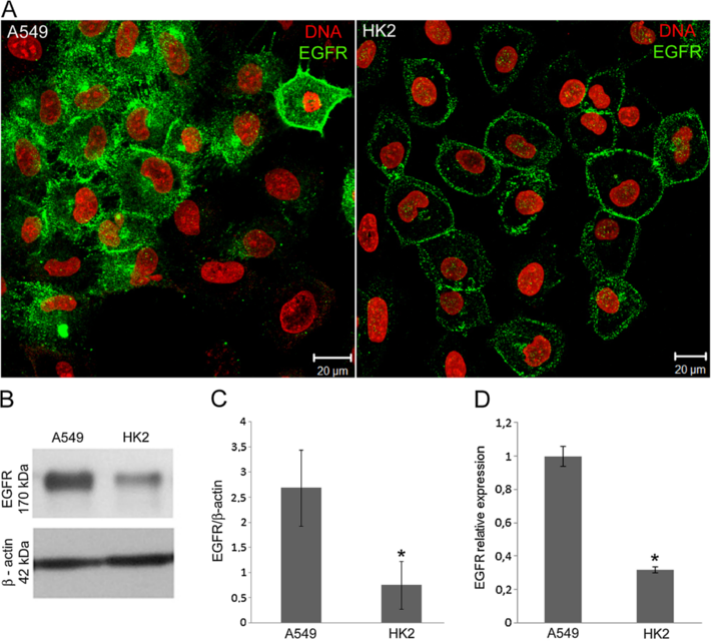
**Cellular localization, expression and mRNA levels of EGFR.** (**A**) Immunofluorescence was performed with an antibody against EGFR (green). The nuclei were stained with propidium iodide (red). EGFR was identified at the cell membrane of both cell types and in clusters near the nucleus in A549 cells. (**B**) EGFR expression in A549 and HK2 cells by Western blotting. (**C**) Quantification of EGFR expression. (**D**) RT-PCR quantification of mRNA levels transcribed by the ErbB1 gene. All results are representative of three or more independent experiments. *p ≤ 0.05. Bars = standard deviation.

The number of ErbB1 gene copies was analyzed in one hundred A549 and HK2 cell nuclei using the FISH method with a dual staining probe for this gene and centromere 7. The centromere 7 labeling, localized near the ErbB1 gene on chromosome 7, was also quantified. The majority of A549 cells presented 3 ErbB1 copies and 3 centromere 7 labels (Figure 1A). The number of ErbB1 copies ranged from 4 to 9 copies, peaking at 5 to 6 copies per nucleus, in the HK2 cells (Figure 1B). The amount of labeled centromere 7 also varied in the HK2 cells (Figure 1B); some cells presented more copies of centromere 7 than of the ErbB1 genes and some contained more copies of ErbB1 than centromere 7, indicating a heterogenic cell population, which suggests possible chromosome instability. The cellular location of EGFR was also investigated in both cell types by immunofluorescence (Figure 2A). In the A549 cells, EGFR was detected at the cell borders, suggesting its localization on the cell membrane, diffusely in the cytoplasm and in perinuclear clusters. However, in HK2 cells, EGFR labeling was rather detected at the cell borders.

The lower levels of EGFR labeling in the cytoplasm suggest that the HK2 cell line presents a lower concentration of EGFR. Therefore, we investigated whether there were differences in the levels of protein expression. Western blotting experiments demonstrated that the HK2 cells manifested reduced receptor expression levels compared to the A549 cells (Figure 2B and C). Quantitative RT-PCR revealed that levels of ErbB1 messenger RNA were higher in the A549 cells than the HK2 cells (Figure 2D).

### Determination of the cellular localization and activation status of EGFR after EGF stimulation

**Figure 3:**
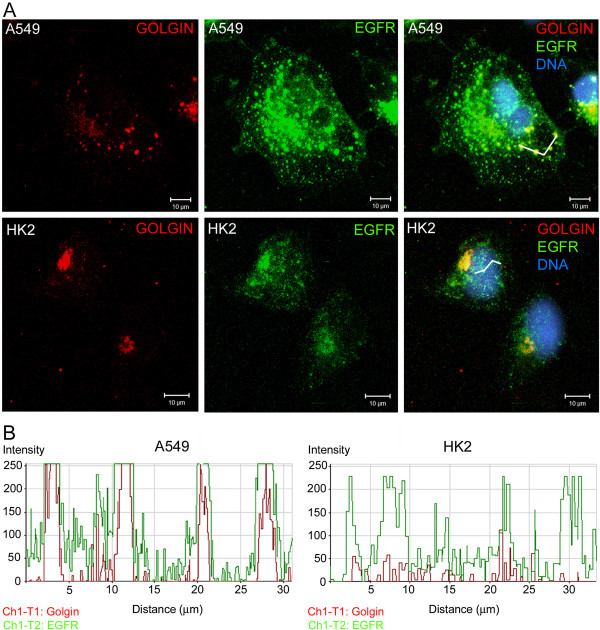
**Detection of the EGFR cellular distribution after EGF stimulation.** (**A**) Cells were cultured in medium containing 10% FCS and treated with EGF (100 ng/ml) for one hour. EGFR (green) was detected in small and numerous vesicle-like agglomerates dispersed in the cytoplasm and in clusters near the nuclei. The Golgi apparatus was detected using an antibody against golgin (red), and the nuclei were stained with DAPI. (**B**) The histograms were generated using the profile display mode tool of LSM 510 version 3.2 software. The co-localization was examined along a trace in a set of combined images. Some vesicle-like structures containing EGFR were co-localized with the Golgi apparatus label. (see also Additional file 1: Figure S1).

A549 cells exhibited significant changes in EGFR distribution after EGF stimulation. The localization of EGFR to the cell borders was altered, and the receptor was located in numerous small agglomerates dispersed in cytoplasm with the appearance of vesicles, and in clusters near the nucleus (Figure 3A). HK2 cells presented some possible cytoplasmic vesicles, but compared to A549 cells, the considerably fewer of these structures were detected (Figure 3A). After EGF stimulation, EGFR was located at the cell borders only in HK2 cells (data not shown).

The Golgi apparatus was detected by immunofluorescence using an antibody against golgin. The histogram in Figure 3B presents the intensity of the green (EGFR) and red (golgin) signals in the cytoplasm at the selected locations and it indicates where signals are co-localized. The EGFR labeling co-localizes with the golgin immunolocalization in the vesicle-like structures in A549 cells, while HK2 cells did not present this co-localization (Figure 3B).

**Figure 4:**
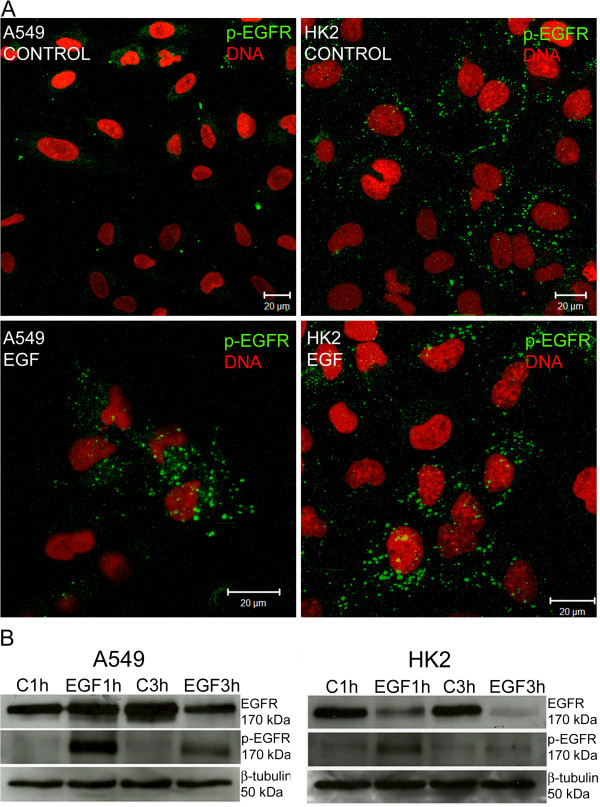
**Location and expression of p-EGFR in A549 and HK2 cell lines.** (**A**) Cells were cultured in medium containing 10% FCS (control) or 10% FCS and EGF (100 ng) for one hour. The labeling for p-EGFR is shown in green, and the nuclei were stained with propidium iodide (red). (**B**) EGFR and p-EGFR proteins were detected by Western blotting, and β-tubulin protein was used as the loading control. The cells were stimulated for one or three hours with EGF (EGF1 h/EGF3 h). The control cells were maintained in medium without EGF. EGFR expression in A549 and HK2 control cells could not be compared because of different time of film exposure.

The phosphorylated form of EGFR (p-EGFR) was analyzed by immunofluorescence in control and EGF-stimulated cells. The A549 control cells did not present p-EGFR labeling, but in the EGF-stimulated cells, p-EGFR could be identified at vesicle-like structures in the cytoplasm (Figure 4A). The pattern of p-EGFR labeling was similar to that of EGFR after EGF stimulation (Figure 3A), suggesting that some of the vesicle-like structures in EGF-stimulated cells likely contain p-EGFR. The HK2 cells presented a different pattern: the phosphorylated form of EGFR was at the borders of control cells and inside vesicle-like structures in EGF-stimulated cells (Figure 4A).

Western blotting was performed to evaluate the total levels of EGFR (intracellular and cell surface) and p-EGFR (Figure 4B). A549 cells maintained the same levels of EGFR 1 hour after stimulation (EGF 1h) compared to control group. After 3 hours of EGF stimulation (EGF 3h) the level of EGFR decreased expression in A549 cells. The presence of the p-EGFR was not detected in control A549 cells (C 1h and C 3h), but after one hour of stimulation (EGF 1h), the p-EGFR was detected, and after three hours of EGF exposure (EGF 3h), the p-EGFR level was observed to be decreased.

HK2 cells showed different responses to the same treatment of EGF stimulation. A severe reduction of EGFR was verified even after one hour of EGF stimulation. However, p-EGFR protein was ready detected in control cells (C1h and C3h). Additionally, after one hour of EGF stimulation, it was possible to observe increased levels of the p-EGFR protein, which subsequently decreased after this timepoint.

All together the results indicate different pathways of EGFR after EGF stimulation. In A549 cells the synthesis of new molecules of EGFR would compensate the degradation of receptors after internalization. This was not observed in HK2.

### EGFR activation by EGF did not induce cell proliferation

Once we demonstrated that EGFR is phosphorylated by EGF exposure in A549 and HK2 cells, the next step was to determine if the EGFR activation contribute to cell proliferation. Both cell lines were submitted to serum starvation (2.5% FCS) followed by EGF addition, and the cell numbers were determined after 24, 48 and 72 hours.

**Figure 5:**
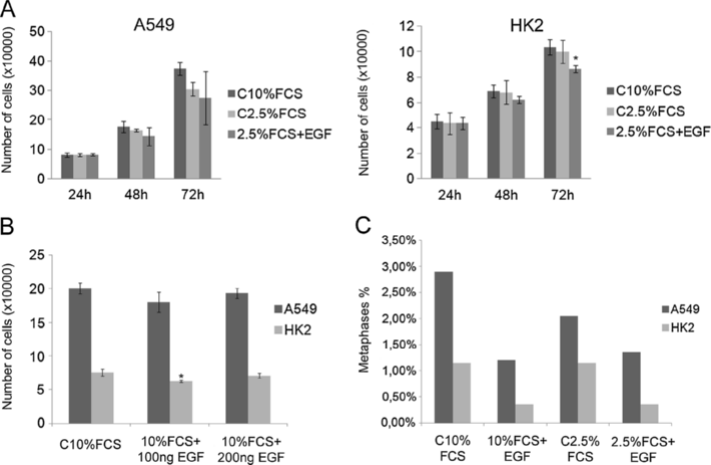
**Determination of cell numbers after EGF stimulation.** (**A**) A549 and HK2 cells were cultured in medium containing 10% FCS (control), 2.5% FCS or 2.5% FCS plus EGF (100 ng), and the cells were counted after 24, 48 and 72 hours. Five thousand cells from each group were analyzed in all experiments. (**B**) The cells were cultured in medium containing 10% FCS with or without EGF (100 or 200 ng) supplementation. The cells were counted after 48 hours. In all experiments, 5000 cells from each group were analyzed. (**C**) The cells were fixed after 48 hours of treatment (C10%FCS, 10%FCS + 100ngEGF, C2.5%FCS, 2.5%FCS + 100ngEGF), and the metaphases were quantified in a total of 2000 cells. All results are representative of three or more independent experiments. *p ≤ 0.05. Bars = standard deviation.

There was no significant difference in the number of cells between the groups of A549 cells at the examined times (Figure 5A). The data analyses using Minitab 15 software indicated a tendency towards decreased cell number in the stimulated group (2.5% FCS + EGF) after 48 and 72 hours. Similar results were observed in the HK2 cell line, no differences in the cell number were detected. However, after 72 hours, the 2.5% FCS + EGF condition was associated with the least number of cells compared to the control group (Figure 5A).

The cell cycle was analyzed in all groups in both cell lines by flow cytometry, and the results are presented in Table 1. The A549 cells stimulated with EGF presented a slight increase in the frequency of cells in the G2/M phase compared to the control cells. The C2.5% FCS condition corresponded to 23.79% of cells in the G2/M phase at 48 hours and 21.55% at 72 hours. The cells treated with EGF (2.5% FCS + EGF) presented 25.59% of cells in G2/M at 48 hours and 24.05% at 72 hours. A decrease in the G1 phase cell population was also observed in the EGF-stimulated A549 cells; 56.14% of the stimulated cells at 48 hours and 58.79% at 72 hours were in G1 compared to 59.52% and 64.51% of control cells at 48 and 72 hours, respectively. However, in HK2 cells, the results indicated that the EGF-stimulated cells exhibited a small decrease in the G2/M phase cell population compared to the control cells. In the 2.5% FCS group, 26.78% of cells were in G2/M at 48 hours and 26.50% at 72 hours. Of the EGF-stimulated cells, 23.47% were in G2/M at 48 hours and 23.06% at 72 hours. A consistent increase in the frequency of G1 cells was also observed in the EGF-stimulated groups, corresponding to 49.58% of cells in G1 at 48 hours and 51.03% at 72 hours in the stimulated cells compared to 46.40% and 42.60% of control cells at 48 and 72 hours, respectively. There were no significant changes in S phase in the groups analyzed.

**Table 1:** Cell cycle analysis in A549 and HK2 cell lines after EGF (100ng) stimulation for 24, 48 and 72 hours

			**24h**			**48h**			**72h**	
**Cell**	**Cycle phase**	**C10% FCS**	**C2.5% FCS**	**2.5% FCS + EGF**	**C10% FCS**	**C2.5% FCS**	**2.5% FCS + EGF**	**C10% FCS**	**C2.5% FCS**	**2.5% FCS + EGF**
	**G1**	52.36%	58.63%	51.8%	57.01%	59.52%	56.14%	60.10%	64.51%	58.78%
**A549**	**S**	13.99%	14.87%	15.42%	12.56%	10.68%	9.72%	10%	9.44%	8.77%
	**G2/M**	24.10%	19.60%	22.64%	24.99%	23.79%	25.59%	23.38%	21.55%	24.05%
	**G1**	41.70%	45.70%	40.94%	43.66%	46.40%	49.58%	46.19%	42.60%	51.03%
**HK2**	**S**	24.45%	22.37%	25.82%	22.55%	21.86%	20.74%	21.94%	17.91%	17.26%
	**G2/M**	39.75%	27.98%	28.78%	29.16%	26.78%	23.47%	27.54%	26.50%	23.06%

To confirm whether the absence of proliferation stimulus in response to the EGF was attributable to serum deprivation, another experiment was designed. Instead of serum starvation conditions, the cells were cultured in the presence of 10% FCS containing 100 or 200 ng/mL EGF, and the number of cells was quantified after 48 hours. The addition of EGF to the 10% FCS medium did not increase cell proliferation compared to the control cells cultured in 10% FCS medium alone (Figure 5B). These data are similar to the results presented in Figure 5A. The cell cycle was also analyzed after 48 hours of EGF stimulation, and the results (Table 2) were similar to those described above. The frequencies of A549 cells in the G1 phase were 46.81% of the C10% cells, 41.28% of the 10%FCS + 100ngEGF cells and 42.10% of the 10%FCS + 200ngEGF cells. Again, the EGF-stimulated cells exhibited a decrease in the frequency of cells in G1. The frequencies of cells in the G2/M phase were 21.92% of the control cells, 22.60% of the 10%FCS + 100ngEGF cells and 23.55% of the 10%FCS + 200ngEGF cells. In the HK2 cells, 38% of the control cells, 44.53% of the 10%FCS + 100ngEGF cells and 44.2% of the 10%FCS + 200ngEGF cells were in G1, indicating an increase of cells in this phase after EGF treatment. The proportions of HK2 cells in the G2/M phase were 26.74% of the control cells, 26.52% of the 10%FCS + 100ngEGF cells and 28.39% of the 10%FCS + 200ngEGF cells. There were no significant changes in S phase in the groups analyzed.

**Table 2:** Cell cycle analysis in A549 and HK2 cell lines after 48 hours of EGF stimulation (100 or 200 ng/mL)

			**48 hours**	
**Cell**	**Cycle**	**C10%**	**10%FCS**	**10%FCS**
	**Phase**	**FCS**	**+100ngEGF**	**+200ngEGF**
	**G1**	46.81%	4128%	42.10%
**A549**	**S**	14.7%	14.22%	14.21%
	**G2/M**	21.92%	22.60%	23.55%
	**G1**	38%	44.53%	44.2%
**HK2**	**S**	24.56%	23.69%	21.92%
	**G2/M**	26.74%	26.52%	28.39%

The mitotic indexes were determined by counting metaphases in the experimental groups. The frequency of metaphases detected in the groups of A549 cells were 2.9% in C10%FCS, 1.20% in 10%FCS + EGF, 2.05% in C2.5% and 1.35% in 2.5%FCS + EGF. In HK2 cells, the frequencies were 1.15% in C10%FCS, 0.35% in 10%FCS + EGF, 1.15% in C2.5% and 0.35% in 2.5%FCS + EGF. It was observed that the EGF-stimulated groups (2.5%FCS + EGF and 10%FCS + EGF) in all of the analyzed conditions presented fewer metaphases compared to the control groups (C10%FCS and C2.5%FCS) (Figure 5C).

Together, these data show that EGF stimulation is not sufficient to promote cell proliferation through EGFR signaling in the analyzed cell lines.

### AG1478 did not interfere with cell proliferation

**Figure 6:**
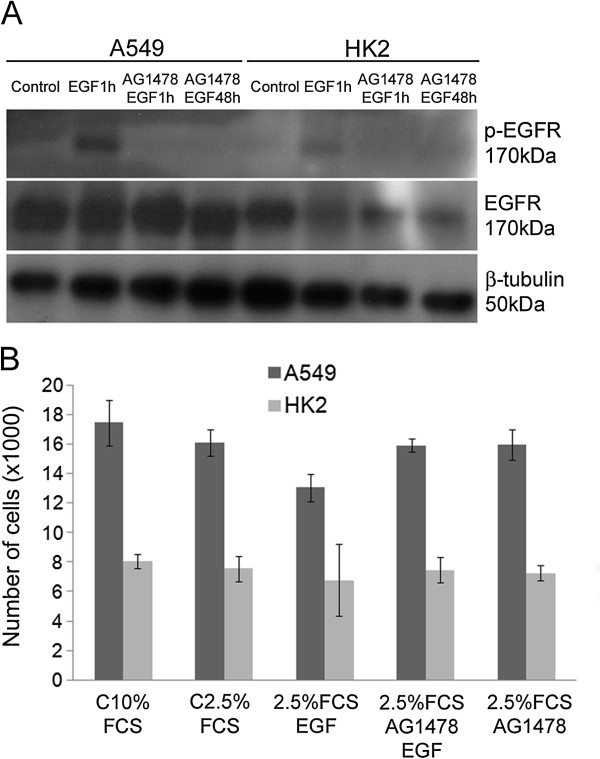
**EGFR inhibition by AG1478 did not interfere with cell proliferation.** (**A**) EGFR inhibition by AG1478 after 1 hour or 48 hours of treatment. (**B**) Determination of the cell numbers after AG1478 inhibition. A549 and HK2 cells were cultured in medium containing 10% FCS, 2.5% FCS, 2.5% FCS plus EGF (100 ng), 2.5% FCS plus AG1478 and EGF or 2.5% FCS plus AG1478. The cells were counted after 48 hours. Five thousand cells from each group were analyzed in all experiments. All results are representative of three or more independent experiments. *p ≤ 0.05. Bars = standard deviation.

The effects of EGFR inhibition on cell proliferation were also evaluated using a tyrosine kinase inhibitor (AG1478). EGFR inhibition was confirmed by western blotting to detect the p-EGFR protein. The p-EGFR protein was absent after 1 hour of treatment, and it remained absent for 48 hours in both cell lines (Figure 6A). The analysis of cell viability using the Via Count (GUAVA Technologies) reagent in flow cytometry indicated that the inhibitor was not cytotoxic to the cells. The A549 control group contained 83.7% viable cells, and the AG1478 inhibited group contained 88.9% viable cells. The HK2 control group contained 79.4% viable cells, and the AG1478 inhibited group contained 83.7% viable cells.

Both cell lines were submitted serum starvation (2.5% FCS) followed by EGF or AG1478 addition, and the numbers of cells were measured after 48 hours. EGFR inhibition did not have an effect on cell proliferation compared to the controls (Figure 6B).

### EGF elicited morphological changes that were suppressed by AG1478

**Figure 7:**
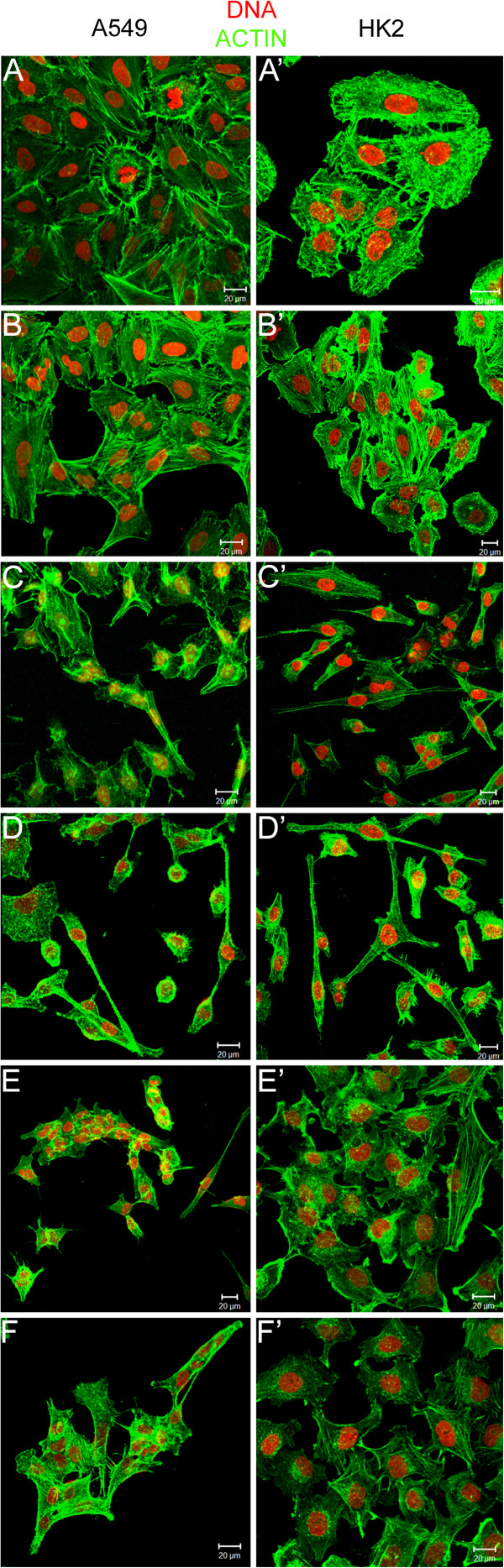
**Evaluation of the effects of EGF on cell morphology.** The control cells (A/A’: C10%FCS, B/B’: C2.5%FCS) were organized into clusters with cell-cell junctions. The EGF (100 ng/ml)-stimulated cells (C/C’: 10%FCS + EGF, D/D’: 2.5%FCS + EGF) manifested fewer adhesions to the surrounding cells, alterations in the organization of actin microfilaments and cytoplasmic protrusions. These alterations induced by EGF were not identified in the cells treated with AG1478 (E/E’: 2.5%FCS + AG1478 + EGF, F/F’: 2.5%FCS + AG1478).

A549 and HK2 cells did not increase proliferation in response to EGF exposure, but some cytoskeletal morphological alterations were detected in the immunofluorescence preparations (Figure 7). The control cells were organized into clusters, characteristic of epithelial cells with cell-to-cell junctions (Figure 7A, A’, B, B’). The EGF stimulation inhibited the organization of HK2 and A549 cells into clusters, and the cells lost the morphological characteristics of an epithelial cell line. The EGF-stimulated cells with altered morphology appeared to have lost cell polarity and exhibited stretching and spreading. Other alterations detected were reduced adhesion to the surrounding cells, altered organization of actin microfilaments and cytoplasmic protrusions (Figure 7C, C’, D, D’). These cytoskeletal alterations were not observed in the cells treated with AG1478 or with AG1478 plus EGF (Figure 7E, E’, F, F’), indicating a potential recovery of epithelial characteristics.

### EGF promoted cell migration

**Figure 8:**
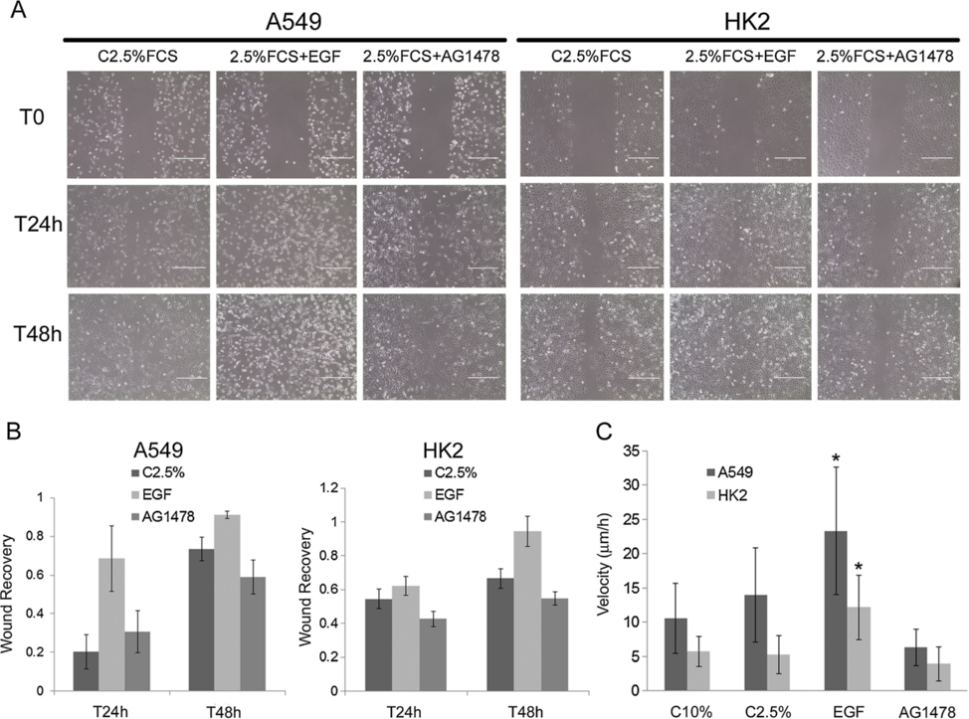
**EGF stimulates cell migration.** (**A**) Wound healing assay was made in both cell lines with 24 and 48 hours of recovery. The groups analyzed were: C2.5%FCS, 2.5%FCS + EGF (100ng/ml), 2.5%FCS + AG1478. Scale bars = 200 μm. (**B**) The graphics present frequency of wound recovery in A549 and HK2 cells. We can observe that EGF-stimulated cells closed the wound faster than control and AG1478 inhibited cells. All results are representative of three independent experiments. *p ≤ 0.05. (**C**) Time-lapse microscopy experiments performed with no directional stimulus for cell migration. It was quantified the velocity of 30 cells of each treatment group, considering the route of the cells during 12 h. EGF treatment stimulated cell migration in both cell lines. Bars = standard deviation.

We also investigated whether the morphological changes elicited by EGF could contribute to cell migration using a wound-healing assay and time lapse microscopy. Wound-healing assay demonstrated that EGF stimulated motility in the A549 and HK2 cells (Figure 8A and B). After 48 h, A549 and HK2 control cells filled 73.45% and 66.66% of the wound area, respectively and EGF-stimulated A549 and HK2 cells filled 90% and 95% of the wound, respectively. Cells treated with EGFR inhibitor exhibited less motility than control cells, indicating that the enhanced cell motility observed was due to EGFR stimulation (Figure 8B). Also, the wound healing was due to cell migration because EGF treatment did not lead to increased cell proliferation.

Time-lapse microscopy experiments were performed with cells cultured in a low density and with no directional stimulus for cell migration. It was quantified the velocity of 30 interphasic cells of each treatment group, considering the route of the cells during 12 h. EGF treatment stimulated cell migration in both cell lines (Figure 8C). A549 and HK2 cultured in 10% FCS medium exhibited velocities of 10.5 μm/h and 5.7 μm/h, respectively; at 2.5% FCS medium, 14 μm/h and 5.2 μm/h, respectively; after EGF treatment A549 cells showed velocity of 23.3 μm/h and HK2 cells exhibited velocity of 12 μm/h. A549 cells treated with AG1478 showed lower velocity compared to 2.5% FCS control group, but similar to the 10% FCS control group. HK2 cells did not present significant changes in velocity of AG1478 treated cells compared to control groups.

It was observed a considerable standard deviation between velocities in both control and EGF-stimulated cells in the same cell line. This indicates that cell behavior can be heterogeneous among cells in the same culture. Despite the standard deviation, statistical analyses demonstrate that EGF-stimulated cells presented higher velocities compared to control cells.

**Figure 9:**
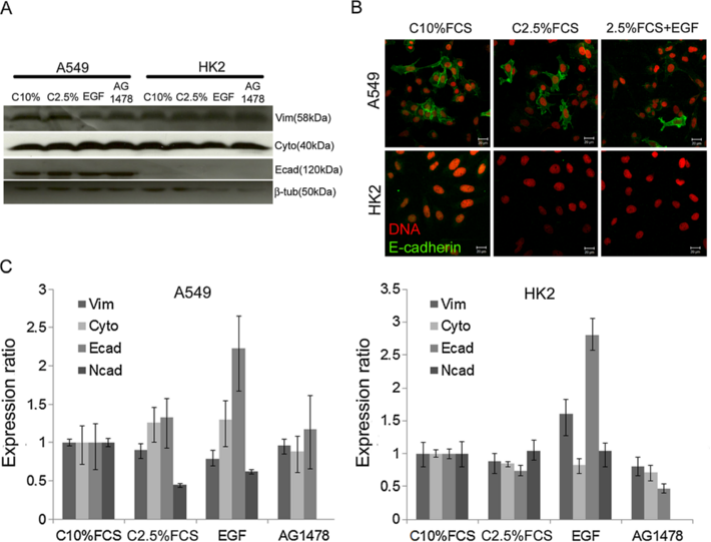
**Analysis of mRNA and protein related to EMT.** (**A**) Vimentin, cytokeratin-18 and E-cadherin expression in A549 and HK2 cells by Western blotting. The groups analyzed were: C10%FCS, C2.5%FCS, 2.5%FCS + EGF and 2.5%FCS + AG1478. Tubulin was used as loading control. (**B**) Immunofluorescence was performed to C10%FCS, C2.5%FCS and C2.5%FCS + EGF groups using an antibody against E-cadherin (green) and the nuclei were stained with propidium iodide (red). (**C**) RT-PCR quantification of mRNA levels of vimentin, cytokeratin-18, E-cadherin and N-cadherin in A549 and HK2 cells. The groups were the same described in **A**. Bars = standard deviation.

Some of the proteins expressed by epithelial or mesenchymal cells were analyzed to investigate whether the EGF-stimulated motility could be due to an epithelial-to-mesenchymal transition (Figure 9A). In A549 cells, no alterations in vimentin, cytokeratin 18 and E-cadherin proteins were observed upon EGF stimulation. HK2 cells exhibited a similar pattern of expression as A549 cells but did not express E-cadherin. The E-cadherin immunofluorescence images support the data obtained by Western blotting (Figure 9B). Positive signal for E-cadherin was observed in A549 cells immunofluorescence, and the protein was detected by western blotting in this cell line. E-cadherin was not detected by immunofluorescence or Western blotting in HK2 cells. The mRNAs encoding these proteins were also evaluated, and alterations related to epithelial-to-mesenchymal transition were not detected (Figure 9C).

N-cadherin is another protein related to epithelial-to-mesenchymal transition, expressed in mesenchymal cells. HK2 cells did not present changes in mRNA levels after EGF stimulation. A549 cells showed a discrete increase in EGF stimulated cells compared to control group cultured in 2.5% FCS (Figure 9C).

## Discussion

We identified 3 ErbB1 gene copies in the A549 cell line, as described previously [17], and heterogeneity in the ErbB1 gene copy number per nuclei in the HK2 cell line. It has been reported that the MDA-MB-468 CD44^+^/CD24^-/LOW^ cell line contains different numbers of ErbB1 copies per nucleus due to the asymmetric segregation of chromosomes during mitosis after the formation of breakage-fusion-bridges [29]. The possibility of a fragile site near the ErbB1 gene has also been suggested to contribute to breaks near the ErbB1 gene region [30]. The HK2 cells are comprised of 2.4% cells undergoing multipolar mitosis and 3.7% micronucleated cells, suggesting that errors in chromosome segregation may occur during mitosis in these cells [31]. Thus, subpopulations of cells with different ErbB1 gene copy numbers were observed in culture. The HK2 cells presented higher genetic instability than the A549 cell line.We also demonstrated that the HK2 cells presented more copies of the ErbB1 gene per nucleus compared to the A549 cells. Previous studies have reported an increased number of ErbB1 gene copies in tumors or cells derived from human lung carcinomas [32,33]. Furthermore, the amount of EGFR was increased in cells with ErbB1 amplifications and mutations [18]. It was expected that the HK2 cells would express more EGFR than the A549 cells, but the opposite was confirmed. The expression of EGFR was decreased due to a reduction in the amount of mRNA transcribed by the ErbB1 gene in the HK2 cells. It is possible that ErbB1 was regulated by methylation, which commonly occurs in certain genes in lung cancer [34].

Many studies have focused on standardization to utilize FISH for patient diagnosis and to determine which patients to treat with EGFR inhibitors. Based on these studies, samples of tumor cells with more than three copies of ErbB1 and gene amplification would be considered FISH positive and indicative of a poor prognosis [35]. Thus, HK2 cells would be potentially FISH positive despite their lower EGFR expression. The level of EGFR expression did not seem to be related to the ErbB1 gene copy number in either cell line.

Beyond the difference in gene copy number, we observed alterations in the EGFR cellular localization and expression level after EGF stimulation. The A549 and HK2 cells contained EGFR in multiple vesicle-like structures in the cytoplasm after EGF treatment. This is in agreement with other studies using A549 [15] and another cell line derived from lung cancer, PC-14 [36].

Activated EGFR is endocytosed by clathrin-coated vesicles, and the receptor can be recycled to the cell membrane or retained in endosomes destined for degradation in lysosomes. The receptor is recycled when the ligand detaches from it. The accelerated internalization removes the activated receptor from membrane, directing it for degradation [10]. The downregulation decreases EGFR levels and cytoplasmic signaling in the cells [37,38]. The vesicle-like structures containing EGFR observed in these two cell lines could be associated with the EGFR degradation pathway, as EGF does not detach from EGFR in the endosomes [10] and stimulates constant receptor internalization [39]. Thus, the vesicle-like structures containing the labeled p-EGFR would be destined for lysosomes.

The vesicles stained with the EGFR and golgin antibodies, observed in A549 cells stimulated with EGF, are most likely involved in the synthesis of this receptor. This finding suggests that the receptors are destined for the cell membrane to replace the degraded EGFR. This EGFR production was not observed in HK2 cells. Supporting these findings, western blotting indicated persistent EGFR levels after EGF stimulation in A549 cells and a decrease in the EGFR levels after EGF stimulation in HK2 cells.

Downregulation can contribute to an increase in the response to therapy with EGFR inhibitors by regulating the effects of EGFR signaling. For example, monoclonal antibodies that bind the EGFR extracellular domain can promote downregulation [40,41], and the endocytosis of EGFR induced by EGF is closely related to the sensitivity to the tyrosine kinase inhibitor gefitinib in non-small cell lung cancer cell lines [42]. These reports suggest that cancer cells that degrade EGFR may be more sensible to treatments. Based on this suggestion, HK2 cells could be considered less aggressive.

The EGFR phosphorylation promoted by EGF did not affect the proliferation of A549 or HK2 cells. Several studies have correlated EGF with proliferation, but the results are contradictory. Previous reports have demonstrated the proliferation of A549 cells after EGF stimulation [43,44]; however, another report did not detect an induction of proliferation by EGF in A549 cells [36]. We found that EGF did not induce cell proliferation, and EGFR inhibition by AG1478 also did not interfere with the number of cells. Stimulation of A549 cells with EGF plus HGF, a c-Met receptor ligand, had a synergistic effect on cell proliferation [45]. It is possible that the EGFR signaling pathway alone was not sufficient to stimulate cell proliferation in a monolayer culture.

It is possible that the AG1478 treatment did not affect the number of cells because of the effects of other tyrosine kinase receptors that continued to signal and promote proliferation. An interaction among different types of tyrosine kinase receptors has been described in glioma; these receptors were co-activated and maintained cytoplasmic signaling. By combining different inhibitors against these receptors, it was possible to achieve a decrease in signaling [46]. The relationship between tyrosine kinase receptors is so close that some lung cancer cell lines exhibited both EGFR and c-Met inhibition after gefitinib treatment [47]. The activation of several tyrosine kinase receptors can potentiate cell signaling, and the inhibition of multiple receptors can more efficiently decrease the response.

Despite the absence of an effect on cell proliferation, EGF-stimulated cells changed their morphology to EMT like [48]. EMT induction by an EGF stimulus has already been described in A549 cells [15,49]. However, alteration in protein expression pattern (low cytokeratin, low E-cadherin and high vimentin) associated with EMT was not detected. Previous works have already demonstrated that A549 and HK2 cells express vimentin despite their epithelial origin [50]. EMT in these cells can occur without an increase in vimentin expression because the protein is already present. E-cadherin expression, other EMT marker was not detected in HK2 cells. A549 cells expressed E-cadherin , but the protein levels did not decrease after EGF treatment as expected. Another study has demonstrated that EMT was induced by EGF in squamous cell carcinomas of the head and neck without changes in E-cadherin expression [51]. Only A549 cells showed a discrete increase in the mRNA levels of N-cadherin in EGF-stimulated cells. EMT cells, in the mesenchymal stage, exhibit cell cycle arrest [48]. Although, there was an increase in G1 cell population in the EGF-stimulated HK2 groups, which can be suggestive of cell cycle arrest. A549 and HK2 cells did not increase proliferation rate after EGF stimulation. It may occur because of cell cycle arrest, what would indicate EMT. Put all together, these data could not support the evidence of EMT.

Despite the absence of evidences of EMT, A549 and HK2 cells showed increased cell motility and cell velocity after EGF stimulation. Wound healing assay and time-lapse analysis also demonstrated that A549 cells presented higher motility compared to HK2 cells and that both cells migrate with and without a migration stimulus. The present results were not enough to confirm EMT by EGF stimulus in A549 and HK2 cells, but they demonstrate that EGF stimulated cell migration in A549 and HK2 cells.

## Conclusion

We have demonstrated that the number of ErbB1 gene copies was not directly correlated with the EGFR expression, in A549 and HK2 cell lines. The activation or inhibition of EGFR did not modify cell proliferation. However, the stimulated cells exhibited morphological changes in the cytoskeleton and increased cell motility.

## Competing interests

The authors declare that they have no competing interests.

## Authors’ contributions

CL and GMMS designed the study. CL, PRT, BAC, GMMS and ELON contributed to experimental procedures and data analysis. All authors critically read, revised and approved the final manuscript.

## Supplementary Material

Additional file 1: Figure S1 Detection of the EGFR cellular distribution after EGF stimulation of A549 cells. Cells were cultured in medium containing 10% FCS and treated with EGF (100 ng/ml) for one hour. EGFR (green) was detected in small and numerous vesicle-like agglomerates dispersed in the cytoplasm and in clusters near the nuclei. The Golgi apparatus was detected using an antibody against golgin (red), and the nuclei were stained with DAPI. Mononucleated cells exhibiting the Golgi apparatus localization similar to HK2 cells. Click here for file
