# Supplementary figures and images for: Independent of ErbB1 gene copy number, EGF stimulates migration but is not associated with cell proliferation in non-small cell lung cancer
Source: Cancer Cell Int. 2013 Apr 30;13:38. doi: 10.1186/1475-2867-13-38 (PMC3655000; doi:10.1186/1475-2867-13-38)

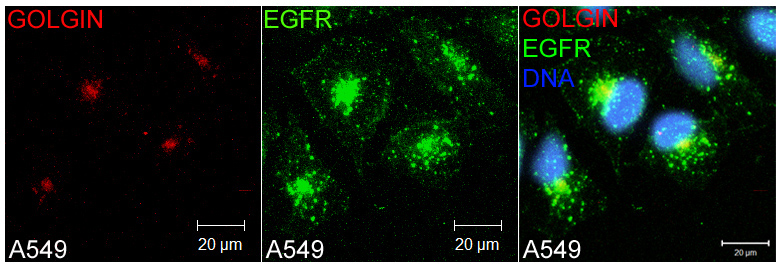

Supplement: Additional file 1: Figure S1 — Detection of the EGFR cellular distribution after EGF stimulation of A549 cells. Cells were cultured in medium containing 10% FCS and treated with EGF (100 ng/ml) for one hour. EGFR (green) was detected in small and numerous vesicle-like agglomerates dispersed in the cytoplasm and in clusters near the nuclei. The Golgi apparatus was detected using an antibody against golgin (red), and the nuclei were stained with DAPI. Mononucleated cells exhibiting the Golgi apparatus localization similar to HK2 cells. [file 1475-2867-13-38-S1.jpeg]
